# Injectable Porcine Collagen in Musculoskeletal Disorders: A Delphi Consensus

**DOI:** 10.3390/jcm14176058

**Published:** 2025-08-27

**Authors:** Orazio De Lucia, Federico Giarda, Andrea Bernetti, Chiara Ceccarelli, Giulia Letizia Mauro, Fabrizio Gervasoni, Lisa Berti, Antonio Robecchi Majnardi

**Affiliations:** 1 Clinical Rheumatology Unit, Department of Rheumatology and Medical Sciences, ASST Centro Traumatologico Ortopedico G. Pini-CTO, 20122 Milan, Italy; oraziodelucia@alice.it; 2 Unit of Rehabilitation Medicine and Neurorehabilitation, Department of Neuroscience, ASST Niguarda Hospital, 20162 Milan, Italy; federico.giarda@ospedaleniguarda.it; 3 Department of Biological and Environmental Sciences and Technologies (DiSTeBA), Università del Salento, 73100 Lecce, Italy; andreabernetti@gmail.com; 4 Department of Rehabilitation, Versilia Hospital, AUSL Tuscany Nordovest, Lido di Camaiore, 55041 Lucca, Italy; chiara.ceccarelli@uslnordovest.toscana.it; 5 Department of Surgery, Oncology and Stomatology, University of Palermo, 90127 Palermo, Italy; giulia.letiziamauro@unipa.it; 6 ASST Fatebenefratelli Sacco, 20131 Milan, Italy; fabrizio.gervasoni@asst-fbf-sacco.it; 7 Physical Medicine and Rehabilitation Unit, IRCCS Istituto Ortopedico Rizzoli, 40136 Bologna, Italy; 8 Department of Biomedical and Neuromotor Sciences, University of Bologna, 40126 Bologna, Italy; 9 IRCCS Istituto Auxologico Italiano, Department of Medicine, Neurology and Rehabilitation, Ospedale San Luca, 55100 Milan, Italy; a.robecchi@auxologico.it; 10 Department of Neurorehabilitation Sciences, IRCCS Istituto Auxologico Italiano, Ospedale San Luca, 20149 Milan, Italy

**Keywords:** Delphi, consensus, porcine collagen injections, musculoskeletal pain, osteoarthritis, tendinopathy, chronic pain, conservative therapy, inflammation, ultra-sound guided injection

## Abstract

Background/Objectives: Musculoskeletal disorders causing chronic pain are increasingly prevalent due to factors such as injury, overuse, and aging, leading to interest in porcine collagen injections as a potential therapeutic and conservative option. Despite promising results, evidence-based information on this treatment is scarce. To address this gap, the authors conducted an eDelphi consensus among expert Italian physicians in musculoskeletal pain to gather their perspectives on collagen injections. Methods: A Steering Committee and a Panel of 23 physicians developed the statements list (36) including the modalities, safety, and efficacy of intra- and extra-articular collagen injections. Panelists rated their agreement with each statement on a 5-point Likert scale (5 means “Strong Agreement”). Consensus was defined as when at least 75% of the panelists voted with a score of ≥4/5 after two rounds of votes. The weighted average (WA) was calculated for each statement. As control, we elaborated a Hypothetical Parametric Distribution (HPD WA equal to 3.00), where the percent of panelists is equally distributed along each Likert Scale Value (LSV). The maximum WA for 75% of the consensus is established at 3.75. Indeed, the combination of 75% having WA > 3.75 was defined as “Strong Agreement”. While, if the consensus was under 75%, the WA vs. HPD comparison was performed using the Wilcoxon Test. Significant differences among the distribution of LSVs judged the statement as “Low Level of Agreement”. Disagreement was evaluated when the WA was under the PHD. Results: The consensus was reached “Strong Agreement” after twin rounds in 29 out of 36 (8.55%). In 5 out of 36 statements (13.89%), the panelists reached the “Low Level of Agreement” by statistical tests. In the remaining two statements, there was a “Consensus of Disagreement”. All panelists unanimously agreed on crucial points, such as contraindications, non-contraindication based solely on comorbidity, and the importance of monitoring collagen’s effectiveness. Unanimous agreement was reached on recommending ultrasound guidance and associating collagen injections with therapeutic exercise and physical modalities. Substantial consensus (concordance > 90%) supported collagen injections for osteoarthritis, chondropathy, and degenerative tendinopathies, emphasizing intra- and peri-articular treatment, even simultaneously. However, areas with limited evidence, such as the combination of collagen with other injectable drugs, treatment of myofascial syndrome, and injection frequency, showed disagreement. The potential of intra-tendinous porcine collagen injections for tendon regeneration yielded mixed results. Conclusions: Clinicians experts in musculoskeletal pain agree on using collagen injections to treat pain originating from joints (e.g., osteoarthritis) and periarticular (e.g., tendinopathies).

## 1. Introduction

Musculoskeletal disorders encompass a wide range of disorders affecting various structures, such as joints, ligaments, tendons, and cartilage, often causing chronic pain and limitation. In recent years, there has been a significant increase in the prevalence of musculoskeletal disorders due to an aging population, overuse, and injuries [1].

Various injection techniques have been explored to treat musculoskeletal complaints, including corticosteroid injections, hyaluronic acid (HA), platelet-rich plasma, and prolotherapy [2]. There is also a growing interest in collagen injections as a potential therapeutic option [3,4,5]. In fact, collagen, the main structural protein in the extracellular matrix, plays a crucial role in tissue integrity and function [6]. Different types of collagens have specific functions in different tissue types [7]. Experimental models using collagen have shown favorable biocompatibility, biodegradability, and weak immunogenic reactions [8]. Porcine injections have been widely used in clinical settings, acting as natural bio-scaffolds and stimulating endogenous collagen synthesis [4,9]. Indeed, porcine collagen, structurally similar to human collagen, is preferred for treating musculoskeletal conditions as well: great trochanteric pain syndrome (GTPS) [10,11], Knee Osteoarthritis (K-OA) [12,13,14], hip tendinitis [15,16,17], rotator cuff disease [15] due to its high safety and low immunogenicity [18] compared with the other most common extraction source of collage, namely bovine collagen [19], making it an ideal material for bio-scaffold production [4,5,20,21] and to be more compatible for humans instead of the bovine one [18]. Indeed, Porcine collagen does not cause allergic reactions unlike bovine collagen, which causes adverse reactions in 3% of the population [18]. Nonetheless, the porcine collagen injection featured tenocytes, demonstrating its relationship with mechanical stimuli [10,11].

Despite the promising results of collagen injections, there is a lack of specific guidelines for these injections in treating musculoskeletal complaints regarding different anatomical regions [10,16,17,21,22], therapeutic indications [16,23,24,25], treatment protocols [2,4,15,26], injection modality [11,26,27], and the combination with other treatments [14,23,28,29]. In order to address this void, a Scientific Board comprising Italian authorities on the diagnosis and treatment of musculoskeletal disorders, launched work on a consensus document. This eDelphi consensus aimed to gather experiences and opinions from Italian physicians, mainly physiatrists and rheumatologists, with expertise in diagnosing and treating musculoskeletal pain. In order to ensure informed clinical practice based on a corpus of knowledge, the aim was to set the path for future guidelines in this area.

## 2. Materials and Methods

### 2.1. Delphi Survey and Panel Composition

**Figure 1:**
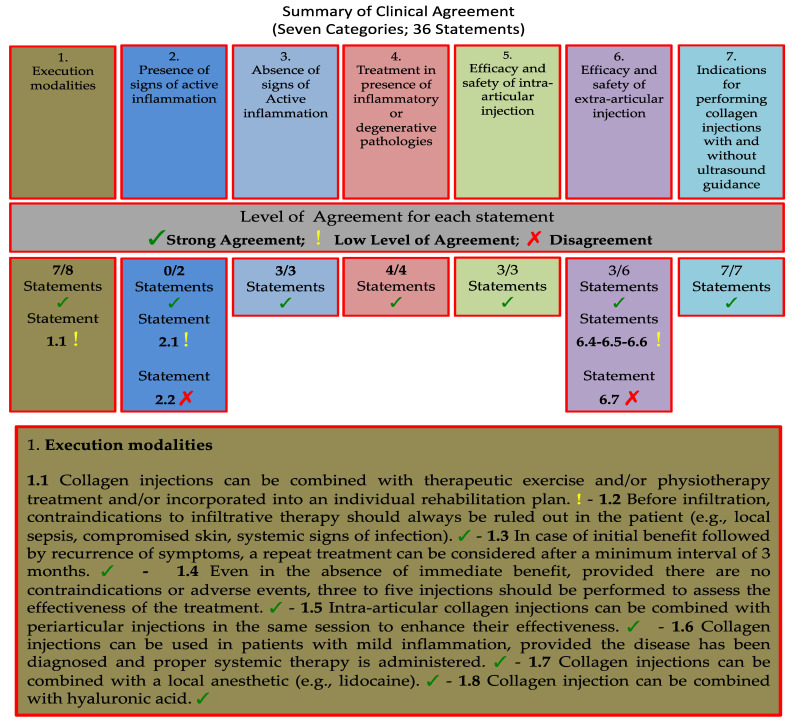

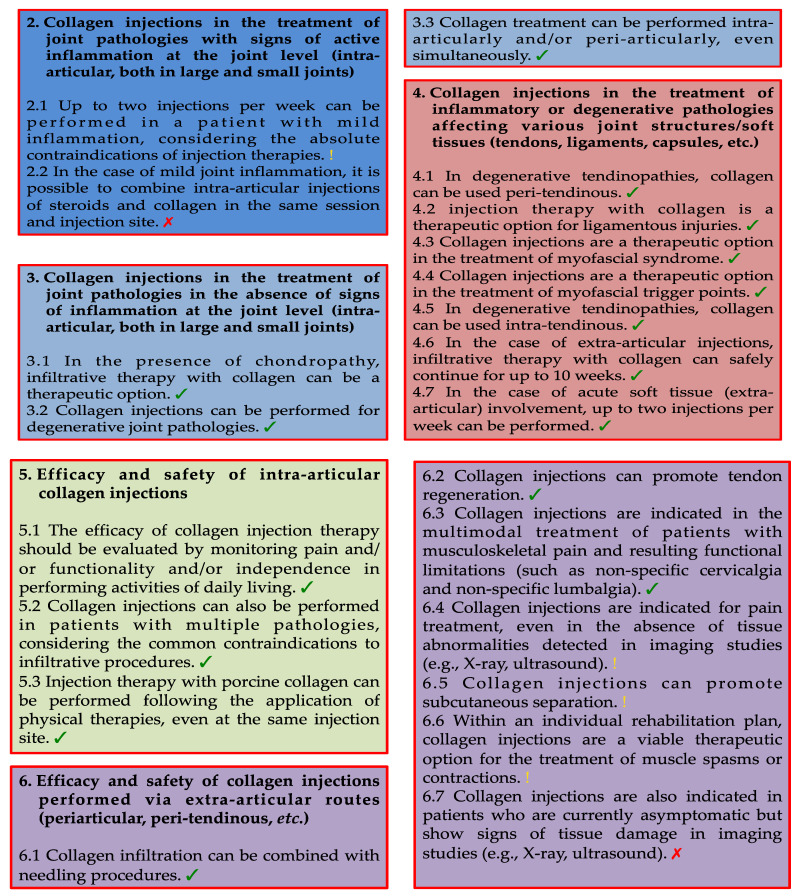

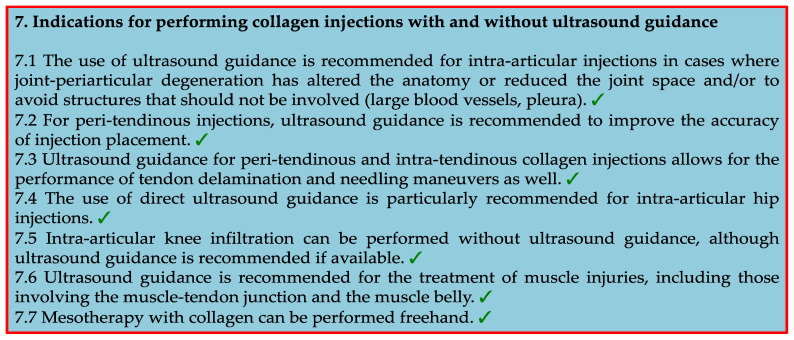
Summary of Clinical Agreement. All 36 statements are represented by different colors, within their respective boxes. The percentages of strong agreement are reported below the box related to the respective category. Each statement is marked according to the legend associated with the image. Additional final considerations regarding the statements without “Strong Agreement” are reported in the last dark gray box.

In this study, we employed a two-round eDelphi process conducted through an online survey aiming to achieve consensus on the use of collagen injections in treating musculoskeletal pain, similar to a previous report by Bernetti et al. [30], aimed at reaching consensus on the use of slow-acting drug for osteoarthritis treatment (SYSADOA) Regarding the use of collagen injections in this specific area, there are no evidence-based guidelines or recommendations. The Steering Committee (SC) comprised highly experienced physiatrists and rheumatologists (DLO, GiF, BA, CA, CC, LMG, GeF, BL, RMA) with substantial expertise in diagnosing and treating musculoskeletal pain. The SC then identified a panel of 23 Italian experts based on their extensive publication record or their significant clinical and academic backgrounds. The criteria mentioned above were reported in the back matter of the paper (Appendix A). The SC agreed to structure the study into seven distinct categories and developed statements within each category (Table 1 and Figure 1).

The web-based eDelphi questionnaire was emailed to each panel member (Figure 1) with a secure link using the private platform, following the Checklist for Reporting Results of Internet E-Surveys (CHERRIES) [31]

**Figure 2:**
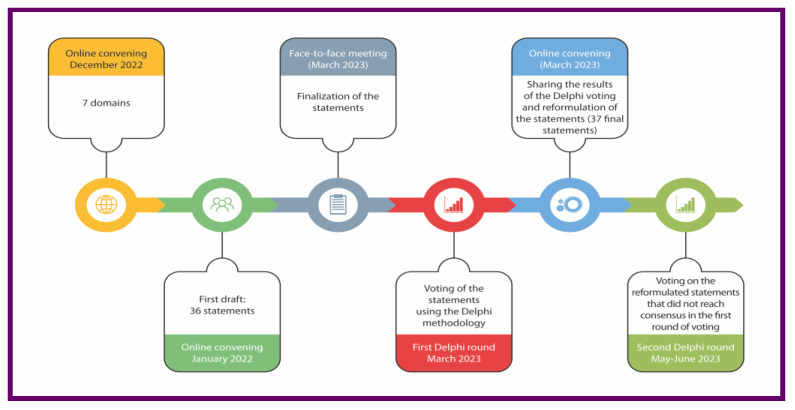
Flowchart depicting the Delphi process employed to establish consensus statements regarding the use of collagen injections in physical and rehabilitation medicine. Source: Original.

Nonetheless, the content of DELHI was performed following the AGREE document acquired from the CREDES guidelines [32] (Figure 2). However, it is important to highlight that CREDES is specific to palliative care and is limited to the Delphi method, which leaves a gap for a reporting guideline that can be applied to other biomedical areas and consensus processes involving non-Delphi-based methods or “modified Delphi” [33].

### 2.2. Opinion Quntification, Threshold Assesment, and Satistical Quntification

**Figure 3:**
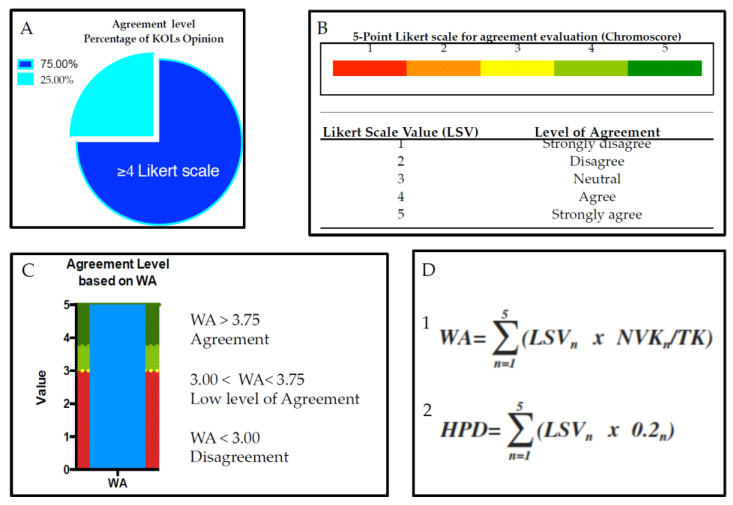
All criteria to evaluate the agreement numbers. (**A**) First criterion of decision for the “Strong Agreement”. (**B**) Likert Scale Values and their associated scores. (**C**) Graph bar regarding the borderline value of WA. (**D**) Equation used in order to obtain the WA of Delphi (equation 1) and the weighted average Hypothetical Parametric Distributions (HPD, equation 2).

The anonymity of their responses was strictly maintained, ensuring that their identities remained undisclosed. Access to the individual responses was only granted to the independent study moderator, who was not affiliated with the SC or the expert Panel. The respondents had to rate their agreement with each statement on a 5-point Likert scale (score 1 indicated “Strong Disagreement”, while score 5 indicated “Strong Agreement”; the score 3 represented the “Neutral” position) [33]. The Likert scale was used jugging the following options for panelists: Strong Disagreement, Disagreement, Neutral, Agreement, Strong Agreement (Figure 3). To facilitate comparisons between different statements within the same category, a Weighted Average (WA) regarding the eDelphi statement was calculated, applying the weighted average formula as well in the equation below (Figure 3D; equation 1). For each statement, the formula considered the product among the Frequency of Key Opinion Leaders voting (NKV) out of the Total Key Opinion Leaders (TK), for the specific value of Likert scale (LSV). The WA for each question was obtained by the sum of all products as reported in equation 1. To understand whether the collected responses were provided randomly or following a causal distribution, a Hypothetical Parametric Distribution (HPD; Figure 3D; equation 2) was calculated assessing the same frequency for each LSV equal to 0.2 (equation 2) for each statement. The WA of HPD was equal to 3.00. This methodology, respond to requirement reported by Murphy et al. [34]. Quantitative analyses were performed comparing WA vs. HPD by Wilcoxon test. Parametric tests were used in order to find statistically significant differences. The results of the comparison mentioned above were judged significant according the *p* values <0.05. Shapiro–Wilk tests were also performed in order to verify the parametric distribution in the three cohorts mentioned above.

**Figure 4:**
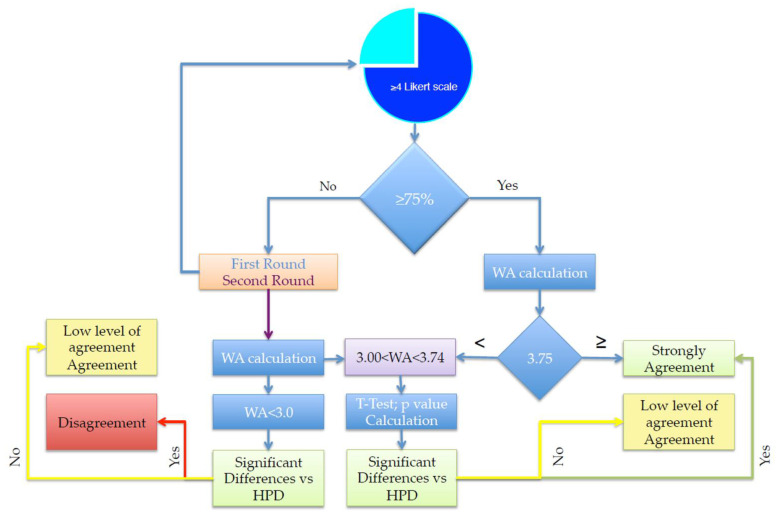
Flowchart for the Agreement decision. The blue BUS indicates the decision three for the agreement. Light green BUS: Algorithm for “Strong Agreement”, Yellow BUS: Algorithm for “Low Level of Agreement”, and Red BUS: “Disagreement”, Orange square: Rounds of decision; Violet square WA assessment.. Two crucial Value foe WA were found: WA > 3.75 means “Strong Agreement”, while WE < 3.00 means “Disagreement”.

Consensus was achieved on a statement when at least 75% of the respondents scored their agreement with the statement as ≥4 of LSV. Notably, the maximum WA in this condition is equal to 3.75%. The SC defines the statement as follows: “Strong Agreement”. When the statement does not reach the condition mentioned above, the following flow chart is used in order to establish which type of consensus is better to assess (Figure 4). 

After the initial phase of the poll, the SC convened virtually to discuss the findings. During this session, they identified statements that did not reach a consensus but were deemed to deserve a revoting when reformulated more precisely. The revised statements were then included in the round 2 (R2) survey. Importantly, all the respondents from the round 1 (R1) survey actively participated in the R2 survey. After the second round of voting, the SC reviewed the panel responses again to assess whether the panelists’ opinions were sufficiently convergent. No ethical approval was required for the present study, as it was limited to collecting clinicians’ opinions and did not involve patient-specific data.

## 3. Results

The total of 23 invited panelists completed the eDelphi questionnaire. All panelists expressed their level of agreement within each statement (no missing items were presented). The R1 eDelphi contained 29 out of 36 statements (80.55%) were judged with “Strong Agreement”, while five statements were revised by the SC and included in the R2 eDelphi. After the second round of voting and the Wilcoxon test, the SC agreed that the panelists’ opinions were sufficiently convergent. These statements (13.89%) were assessed as “Low Level of Agreement” As final report result, two statements (5.56%) received a consensus for a “Disagreement”.

### 3.1. Category 1: Execution Modalities of Collagen Injections in Musculoskeletal Disorders

Within the topic area of the execution modalities of porcine collagen injections in musculoskeletal disorders, the panelists reached a consensus on 7 out 8 statements with a “Strong Agreement”, while the statement C1.1 received a “Low Level of Agreement” (Table 2).

STATEMENTS
1.1 Collagen injections can be combined with therapeutic exercise and/or physiotherapy treatment and/or incorporated into an individual rehabilitation plan.
1.2 Before infiltration, contraindications to infiltrative therapy should always be ruled out in the patient (e.g., local sepsis, compromised skin, systemic signs of infection).
1.3 In case of initial benefits followed by a recurrence of symptoms, a repeat treatment can be considered after a minimum interval of 3 months.
1.4 Even in the absence of immediate benefit, provided there are no contraindications or adverse events, three to five injections should be performed to assess the effectiveness of the treatment.
1.5 Intra-articular collagen injections can be combined with periarticular injections in the same session to enhance their effectiveness.
1.6 Collagen injections can be used in patients with mild inflammation, provided the disease has been diagnosed and proper systemic therapy is administered.
1.7 Collagen injections can be combined with a local anesthetic (e.g., lidocaine).
1.8 Collagen injection can be combined with hyaluronic acid.

A complete consensus was reached (23/23 panelists assigned a score of 4/5 or 5/5 to their agreement level with the statements) on the association of collagen injections with therapeutic exercise and physiotherapy (Table 2). In addition, the panelists unanimously stressed (100% agreement) the significance of excluding any contraindication to infiltrative therapy such as local sepsis and compromised skin or systemic signs of infection before proceeding with the infiltration procedure (Table 2).

The panel also reached a consensus (20 out of 23; 87%) on the appropriateness of repeating porcine collagen injections in cases where initial benefits were observed, followed by symptom recurrence, provided that at least 3 months have elapsed since the previous treatment (Table 2). In case, the immediate benefit was not observed. A total of 87% (20 out of 23) of the panelists agreed to perform three to five porcine collagen injections to evaluate the effectiveness of the treatment, provided there were no contraindications or adverse events (Table 2). The possibility of combining intra-articular collagen injections with periarticular injections in the same session received a consensus rate of 83% (19 out of 23) (Table 2). The use of porcine collagen injections in patients with mild inflammation provided that the disease was diagnosed, and any systemic therapy was adequately administered achieved (18 out of 23; 78%; Table 2). The panelists had a low level regarding whether porcine collagen injections could be combined with local anesthetic (e.g., lidocaine) (15 out of 23; 65% agreement; Table 2). Furthermore, a low percentage of agreement (7 out of 23; 30%) was reported for the possibility of combining collagen infiltration and HA (Table 2).

### 3.2. Category 2: Collagen Injections in the Treatment of Joint Pathologies with Signs of Active Inflammation at the Joint Level (Intra-Articular, Both in Large and Small Joints)

None of the statements in this area reached a 75% consensus among the panelists (Table 3).

STATEMENTS
2.1 Up to two injections per week can be performed in a patient with mild inflammation, considering the absolute contraindications of injection therapies.
2.2 In the case of mild joint inflammation, it is possible to combine intra-articular injections of steroids and collagen in the same session and injection site.

Only 57% (13 out of 23) of the panelists agreed that up to two collagen injections per week could be performed on a patient with mild inflammation once absolute contraindications of infiltrative therapies have been ruled out (Table 3).

Originally, the statement was formulated as: “Up to two injections per week can be performed to achieve the clinical outcome in a patient with signs of inflammation”; it received 30% agreement in the first Delphi round (7 out of 23). The panelists commented that a corticosteroid infiltration should be performed before initiating collagen injections to reduce inflammation. Some panelists argued that corticosteroids and collagen injections could be alternated within the same week. Additionally, the panelists stressed the importance of specifying the type of inflammation. whether it is arthritis. osteoarthrosis. or trauma. The SC reworded the statement: “Up to two injections per week can be performed in a patient with mild inflammation. taking into consideration the absolute contraindications of infiltrative therapies.” But as reported above, in the second Delphi round, consensus was still not achieved (57% agreement). The statement regarding the possibility of combining intra-articular injections of steroids and porcine collagen in the same session and injection site in cases of mild joint inflammation did not reach agreement (10 out of 23; 43%; Table 3).

### 3.3. Category 3: Collagen Injections in the Treatment of Joint Pathologies in the Absence of Signs of Inflammation at the Joint Level (Intra-Articular. Both in Large and Small Joints)

All three statements regarding the treatment of joint pathologies in the absence of signs of inflammation garnered high consensus (Table 4). The panel achieved high consensus (22 out of 23; 96%) regarding including porcine collagen injections as a therapeutic option for chondropathy (Table 4). Additionally, there was high agreement (21 out of 23; 91%) on the possibility of performing porcine collagen injections for degenerative joint pathologies and injecting collagen both intra-articularly and extra-articularly (peri-articularly), even simultaneously (Table 4).

STATEMENTS
3.1 In the presence of chondropathy, infiltrative therapy with collagen can be a therapeutic option.
3.2 Collagen injections can be performed for degenerative joint pathologies.
3.3 Collagen treatment can be performed intra-articularly and/or peri-articularly, even simultaneously.

### 3.4. Category 4: Collagen Injections in the Treatment of Inflammatory or Degenerative Pathologies Affecting Various Joint Structures/Soft Tissues (Tendons, Ligaments. Capsules, etc.)

Almost unanimous agreement was reached (22 out of 23; 96%) on the appropriateness of peritendinous porcine collagen injections for treating degenerative tendinopathies (Table 5).

STATEMENTS
4.1 In degenerative tendinopathies, collagen can be used as peri-tendinous.
4.2 Injection therapy with collagen is a therapeutic option for ligamentous injuries.
4.3 Collagen injections are a therapeutic option in the treatment of myofascial syndrome.
4.4 Collagen injections are a therapeutic option in the treatment of myofascial trigger points.
4.5 In degenerative tendinopathies, collagen can be used intra-tendinous.
4.6 In the case of extra-articular injections, infiltrative therapy with collagen can safely continue for up to 10 weeks.
4.7 In the case of acute soft tissue (extra-articular) involvement, up to two injections per week can be performed.

Seventy-eight percent of the panelists (18 out of 23) considered infiltrative therapy with collagen as a therapeutic option for ligamentous injuries (Table 5). However, the use of collagen injections for the treatment of myofascial syndrome and trigger points did not reach the consensus threshold. Although the percentages of agreement were relevant (74% [17 out of 23] and 70% [16 out of 23], respectively; Table 5). Originally, myofascial syndrome and trigger points were included in a single statement. but this did not reach consensus in the first Delphi round (17 out of 23; 74%). The panelists expressed that they would have rated the use of porcine collagen injections for myofascial syndrome and trigger points differently. As a result, the SC decided to split the statement into two separate statements, one for each condition. However, even in the second round, these statements did not reach the 75% agreement threshold (74% agreement for myofascial syndrome and 70% for trigger points-related statements, respectively). The panelists did not reach a consensus on the use of collagen intra-tendinous in the case of degenerative tendinopathies (15 out of 23; 65%) (Table 5). In relation to extra-articular injections, there was no consensus on the possibility of safely continuing porcine collagen injection therapy for up to 10 weeks (13 out of 23; 57%; Table 5). Similarly, the possibility of performing up to two injections per week in the case of acute soft tissue involvement (12 out of 23; 52% agreement) did not reach consensus (Table 5).

### 3.5. Category 5: Efficacy and Safety of Intra-Articular Collagen Injections

The Panelists unanimously agreed on the importance of evaluating the effectiveness of porcine collagen injection therapy by monitoring pain function and independence in performing activities of daily living (Table 6).

STATEMENTS
5.1 The efficacy of collagen injection therapy should be evaluated by monitoring pain and/or functionality and/or independence in performing activities of daily living.
5.2 Collagen injections can also be performed in patients with multiple pathologies, considering the common contraindications to infiltrative procedures.
5.3 Injection therapy with porcine collagen can be performed following the application of physical therapies, even at the same injection site.

They also unanimously agreed on the possibility of performing porcine collagen injections in patients with multiple pathologies while considering the before-mentioned common contraindications to infiltrative procedures (Table 6).

Furthermore, 83% of the Panelists (19 out of 23) agreed on the possibility of performing porcine collagen injection therapy following the application of physical therapies, even at the same injection site (Table 6).

### 3.6. Category 6: Efficacy and Safety of Collagen Injections Performed via Extra-Articular Routes (Periarticular, Peri-Tendinous, etc.)

Only one statement within this category reached consensus, which was the possibility of combining porcine collagen injections with needling procedures (19 out of 23; 83%; Table 7).

STATEMENTS
6.1 Collagen infiltration can be combined with needling procedures.
6.2 Collagen injections can promote tendon regeneration.
6.3 Collagen injections are indicated in the multimodal treatment of patients with musculoskeletal pain and resulting functional limitations (such as non-specific cervicalgia and non-specific lumbalgia).
6.4 Collagen injections are indicated for pain treatment, even in the absence of tissue abnormalities detected in imaging studies (e.g., X-ray, ultrasound).
6.5 Collagen injections can promote subcutaneous separation.
6.6 Within an individual rehabilitation plan, collagen injections are a viable therapeutic option for the treatment of muscle spasms or contractions.
6.7 Collagen injections are also indicated in patients who are currently asymptomatic but show signs of tissue damage in imaging studies (e.g., X-ray, ultrasound).

The panel disagreed with the affirmation that collagen injections can promote tendon regeneration (15 out of 23; 65%; Table 7). Only 65% of the panelists (15 out of 23) believed that collagen injections are indicated in the multimodal treatment of patients with musculoskeletal pain and resulting functional limitations (such as non-specific cervicalgia and non-specific low back pain) (Table 7). The statement was revised after the first Delphi round: the original statement was “collagen injections are recommended in cases where the patient complains of musculoskeletal pain and reduced mobility (e.g., in the cervical spine)” and received an agreement rate of 52% (12 out of 23). The statement was reworded by the SC as follows: “Collagen injections are indicated in the multimodal treatment of patients with musculoskeletal pain and resulting functional limitations (such as non-specific cervicalgia and non-specific low back pain).” But it still did not reach the threshold for agreement in the second round. The statement regarding the use of collagen injections for pain treatment even in the absence of tissue abnormalities detected in imaging studies (e.g., X-ray. ultrasound) did not reach an agreement (13 out of 23; 57%) (Table 7). Similarly, the use of collagen injections in asymptomatic patients who show signs of tissue damage in imaging studies did not reach consensus (8 out of 23; 35%; Table 7). Only 52% of the panelists (12 out of 23) agreed that collagen injections can promote subcutaneous tissue plane separation (Table 7). The original statement: “Collagen injections are recommended in cases where the patient presents muscle spasms or contractures (e.g., in the cervical or lumbar spine)” reached 43% agreement (10 out of 23), with one panelist commenting that there are likely no side effects but expressing skepticism about their beneficial effects. The SC revised the statement to “Within an individual rehabilitation plan. collagen injections are a viable therapeutic option for the treatment of muscle spasms or contractions.” However, the percentage of agreement achieved upon revoting during the second Delphi round was only 48% (11 out of 23).

### 3.7. Category 7: Indications for Performing Collagen Injections with and Without Ultrasound Guidance

All the statements within this category have achieved consensus, indicating that clinicians prefer to utilize ultrasound guidance whenever available while performing collagen injections (Table 8).

STATEMENTS
7.1 The use of ultrasound guidance is recommended for intra-articular injections in cases where joint-periarticular degeneration has altered the anatomy or reduced the joint space and/or to avoid structures that should not be involved (large blood vessels, pleura).
7.2 For peri-tendinous injections, ultrasound guidance is recommended to improve the accuracy of injection placement.
7.3 Ultrasound guidance for peri-tendinous and intra-tendinous collagen injections allows for the performance of tendon delamination and needling maneuvers as well.
7.4 The use of direct ultrasound guidance is particularly recommended for intra-articular hip injections.
7.5 Intra-articular knee infiltration can be performed without ultrasound guidance, although ultrasound guidance is recommended if available.
7.6 Ultrasound guidance is recommended for the treatment of muscle injuries, including those involving the muscle-tendon junction and the muscle belly.
7.7 Mesotherapy with collagen can be performed freehand.

The Panelists agreed on the recommendation to use ultrasound guidance in the following scenarios (Table 8):

− For intra-articular injections when there are alterations in the joint space anatomy or dimensions or to avoid structures that should not be involved (e.g., large blood vessels of pleura; 23 out of 23; 100% agreement);
− For peri-tendinous injections to improve the accuracy of injection placement (22 out of 23; 96%);
− To perform tendon delamination and needling maneuvers in both peri-tendinous and intra-tendinous collagen injections (22 out of 23; 96%);
− To perform intra-articular hip injections (22 out of 23; 96%);
− For the treatment of muscle injuries, including those involving the muscle-tendon junction and the muscle belly (20 out of 23; 87%).

The panelists thought that ultrasound guidance is highly recommended when available to enhance injection accuracy and ensure precise intra-articular joint space targeting (22 out of 23; 96%; Table 8). According to the panel, collagen mesotherapy can be performed freehand, meaning without ultrasound guidance (20 out of 23; 87%; Table 8). This statement was originally formulated as “The administration of medication through mesotherapy does not require ultrasound guidance” and failed to reach a consensus, even if not by a long shot (17 out of 23; 74%). Therefore, the SC decided to reformulate it to “Mesotherapy with collagen can be performed freehand.” It was revoted in the second Delphi round and reached the threshold for consensus (Table 8).

## 4. Discussion

This Delphi study aimed to gather expert opinions about administering porcine collagen injections in musculoskeletal pain. The study was structured around seven distinct categories that spanned a broad spectrum of considerations related to porcine collagen injections. Within these categories, 37 statements were crafted, each intended to explore specific facets of the procedure. These statements addressed crucial aspects such as execution modalities, safety, efficacy, and the potential benefits of administering ultrasound-guided collagen injections. Upon completing the Delphi process, the expert panel reached a consensus on 22 of the 37 statements. The following sections delve into this Delphi survey’s key findings and implications. These results contribute significantly to understanding the current use of porcine collagen injections in clinical practice in musculoskeletal pain management also critical conditions as demonstrated during the pandemic period [35]. In addition, they also provide a foundation for further research.

### 4.1. Category 1: Execution Modalities of Collagen Injections in Musculoskeletal Disorders

The experts unanimously believed collagen injections could be fruitfully combined with therapeutic exercise or physiotherapy and integrated into an individual rehabilitation plan. In this respect, their experience confirms the literature data showing some beneficial effects of the combination of rehabilitative treatment and collagen injections in treating lumbar pain [36]. Supporting the advantages of combining injection therapy and physiotherapy. The combination of HA injections and physiotherapy proved effective in rehabilitating patients with moderate knee osteoarthritis [32].

The panelists unanimously agreed that absolute contraindications to collagen injections are generally consistent with those for any therapeutic injection procedure, including drug allergies and infections [33].

In total, 35% of the experts disagreed with combining collagen and local anesthetic injections, and the related statement did not reach a consensus. Specific concerns were raised regarding mixing collagen and anesthetic in the same syringe. Despite the limited evidence available in the literature supporting the use of collagen and lidocaine, some members of the SC pointed out that lidocaine could be beneficial in addressing pain or alleviating discomfort resulting from the distension of a particularly constricted target space after collagen injection. Additionally, they proposed that collagen could be a valuable adjunctive therapeutic option in clinical settings where lidocaine injections are already employed such as in treating trigger points [5]. Furthermore, it should be noted that combined injections of porcine collagen and lidocaine have previously been administered in a case series of patients with Morton’s neuroma with no reported side effects [23]. Combining collagen injections with HA injections drew disagreement among 70% of the panelists. Some panelists felt that specific clinical experience was needed or stressed the lack of relevant literature demonstrating the efficacy of this combination. Others emphasized the importance of avoiding both infiltrations in the same session, using the same syringe or of the same anatomical district. Some early clinical findings suggest potential benefits from combining collagen and HA injections: in in vivo studies. HA injections have shown the ability to slow osteoarthritis progression [34] by reducing glycosaminoglycan release and pro-inflammatory molecules, such as MMP-13, MMP-3, and IL-1β [35,37]. Porcine type I collagen increases the expression of tissue inhibitors of metalloproteinase-1 and leads to an augmentation in collagen type I secretion in cultured human tenocytes [9]. This effect is likely favored by the inhibition of collagen degradation by MMP-1 [9]. A non-inferiority prospective randomized controlled trial by Martin et al. observed benefits in patients treated with intra-articular injections of porcine type I collagen or sodium hyaluronate [4]. Collagen injected peri-articularly may stabilize the extracellular matrix. as suggested by Randelli et al. [9,38]. As early as a decade ago, Matsiko and colleagues [39] demonstrated that incorporating HA into collagen scaffolds stimulated the migration and chondrogenesis of mesenchymal stem cells. Similar results have been obtained recently by Muran et al. [40]. The potential synergistic effect of HA and collagen injections makes this a promising clinical avenue for future studies.

### 4.2. Category 2: Collagen Injections in the Treatment of Joint Pathologies with Signs of Active Inflammation at the Joint Level (Intra-Articular. Both in Large and Small Joints)

Forty-three percent of the experts disagreed with the possibility of performing up to two injections per week. In particular, they expressed concerns about the risk of infection. In this line of reasoning, the need to rule out infection before injection was stressed and panelists showed skepticism towards infiltrative therapy when signs of inflammation were present. Similarly, regarding the frequency of collagen injections, a recent review indicates that most studies involving intra-articular collagen injections for knee osteoarthritis administered the injections once a week or less frequently [12]. This frequency may be due to the satisfactory results obtained with this regimen but evidence of the efficacy and safety of increasing the frequency of injections is lacking. In total, 57% of the experts disagreed with combining intra-articular injections of steroids and collagen in the same session and injection site for mild joint inflammation. There are no reports that demonstrate the usefulness and safety of this approach.

Corticosteroid injections are commonly employed to treat joint pathologies characterized by synovial inflammation, such as osteoarthritis [29]. However, the effects of corticosteroids are short-lived, making them more suitable for short-term pain relief [29]. On the other hand, type I collagen injections seem to have a more lasting effect on osteoarthritis than corticosteroids [41].

### 4.3. Category 3: Collagen Injections in the Treatment of Joint Pathologies in the Absence of Signs of Inflammation at the Joint Level (Intra-Articular, Both in Large and Small Joints)

The panel showed a firm consensus (96%) on including porcine type I collagen injections as a therapeutic option for chondropathy. It is noteworthy here that intra-articular collagen injections have the potential to stimulate the production of hyaline cartilage by chondrocytes thus countering cartilage erosion commonly observed in joint pathologies. including osteoarthritis [41]. The experts reached a high consensus (91%) on the possibility of performing porcine collagen injections in the presence of degenerative joint pathologies, aligning with the existing literature suggesting collagen injections’ positive effects in addressing osteoarthritis symptoms and progression [3,4,22,41,42]. Collagen injections showed efficacy in treating musculoskeletal disorders whether administered in the peri-articular [25] or intra-articular space [22,42]. The panelists’ responses showed that both these routes are used in clinical practice. In conclusion, it is important to emphasize the high degree of consensus among the panelists (91%) regarding the simultaneous use of intra-articular and periarticular collagen injections.

### 4.4. Category 4: Collagen Injections in the Treatment of Inflammatory or Degenerative Pathologies Affecting Various Joint Structures/Soft Tissues (Tendons, Ligaments, Capsules, etc.)

The possibility of using porcine collagen injections as therapeutic options for treating myofascial syndrome and myofascial trigger points did not achieve consensus (74% and 70%., respectively), although it approached the threshold of 75% agreement. The pain associated with myofascial syndrome primarily arises from trigger points, which are small nodules, bumps or knots within the muscle that cause pain when compressed [5]. In a study by Nitecka-Buchta et al. [5], the effectiveness of two intramuscular injections, 1 week apart, of porcine type 1 collagen or lidocaine into the trigger points of the masseter muscle in patients with myofascial pain was compared. The study found that collagen injections were more effective than lidocaine in reducing masseter muscle activity and alleviating pain [5]. However, there is controversy about the relative contribution of collagen itself and dry needling in injection therapy for trigger points. Dry needling of myofascial trigger points in the masseter muscle has been reported to provide immediate improvement in pain and jaw function, with effects lasting for at least 1 week [43]. Other studies also support the efficacy of dry needling for treating musculoskeletal pain [44]. Nonetheless, it is believed that while needling per se may contribute to the pain-relieving effect of collagen injections, the long-term benefits, possibly due to its regenerative potential, come primarily from collagen’s biological properties. In total, 35% of the experts disagreed with using intra-tendinous porcine collagen injections in degenerative tendinopathies. One panelist suggested that physical therapies such as ultrasound, magnetic fields, laser or electric stimulation, should be associated with intra-tendinous collagen infiltration for a beneficial response. The efficacy of collagen injections in the treatment of tendinopathies has only recently been investigated. with studies providing valuable insights [17,27,45,46]. Kim et al. [27] conducted the first randomized clinical trial in 2020. They found that a single ultrasound-guided intratendinous atelocollagen injection led to a decrease in tear size and functional improvement in about one out of three patients with partial-thickness rotator cuff tears [27]. A case series reported that intratendinous porcine type I collagen injections improved function and decreased pain in chronic supraspinatus tendinopathies [16].

### 4.5. Category 5: Efficacy and Safety of Intra-Articular Collagen Injections

Eighty-three percent of the panelists agreed with the possibility of performing porcine collagen injection therapy after applying physical therapies, even at the same injection site. Evidence indicates that physical therapies, such as extracorporeal shockwave therapy (ESWT), low-level laser therapy (LLLT) and ultrasound can enhance collagen synthesis and tendon repair after injury [47,48]. Similarly. LLLT, ultrasound and the combined LLLT and ultrasound therapy have increased type I collagen synthesis in animal tendons following injury [49]. On this basis, it can be hypothesized that these physical modalities may work as adjuvant therapies when performing collagen injections.

### 4.6. Category 6: Efficacy and Safety of Collagen Injections Performed via Extra-Articular Routes (Periarticular, Peri-Tendinous, etc.)

Eighty-three of the panelists agreed with the possibility of combining porcine collagen and needling procedures. Needling combined with infiltration is considered an effective modality for immediate pain relief in the management of soft tissue pain and treatment of soft tissue damage [24]. Consensus was not achieved on the potential of porcine collagen injections in promoting tendon regeneration (65% agreement). One panelist suggested that it should be associated with ESWT which, as reported above, can stimulate collagen production [50]. However, several studies support both in vitro and in vivo collagen regenerative potential in tendinopathies [9,21,51,52]. Consensus was also not achieved on the appropriateness of porcine collagen injections for multimodal treatment of patients with musculoskeletal pain and resulting functional limitations (such as non-specific cervicalgia and non-specific low back pain) (65% agreement). The panelists emphasized the need for a specific diagnosis of musculoskeletal pain before providing therapeutic indications. They stated that the appropriateness of collagen injections in this setting depends on several factor, such as comorbidities, ongoing pharmacological therapies, and individual characteristics. Although some panelists believed that collagen injections have limited efficacy in treating cervicalgia and low back pain others acknowledged that collagen injections can still be considered an additional therapeutic opportunity.

The panel’s responses showed high disagreement on the appropriateness of porcine collagen injections for pain treatment in the absence of tissue abnormalities detected by imaging studies (57% agreement), and for the treatment of asymptomatic patients who exhibit signs of tissue damage in imaging studies (35% agreement). Moreover, some panelists stressed that minimally invasive procedures should not be performed without a definitive diagnosis and identification of the source of pain. The appropriateness of collagen injections as viable therapeutic options for the treatment of muscle spasms or contractures also encountered high disagreement (48% agreement). Some panelists expressed support for the adjunctive use of collagen injections in conjunction with other therapeutic approaches but only as a second-line treatment for muscle spasms or contractions.

### 4.7. Category 7: Indications for Performing Collagen Injections with and Without Ultrasound Guidance

Finally, the experts underlined the importance of using ultrasound guidance when performing porcine collagen injections in various conditions (>85% agreement for all the statements). Ultrasound imaging is considered the preferable modality for guiding the majority of interventional musculoskeletal procedures. Unlike other imaging techniques ultrasound provides the distinct advantage of visualizing soft tissues. bony structures. and the needle in real-time [53]. This real-time scanning capability allows for precise guidance and accurate needle placement. Furthermore, ultrasound imaging has no known contraindications (e.g., it does not use ionizing radiation), making it a safe option for guiding musculoskeletal interventions [53]. Ultrasound guidance is strongly recommended for intra-articular injections into the hip joint because of several factors [54]. The depth of the hip joint. combined with the absence of palpable anatomical landmarks, makes it challenging to perform accurate injections without imaging guidance [54]. Additionally, crucial neurovascular structures along the needle path, including the femoral nerve and vessels, increase the risk of potential complications. Therefore, ultrasound guidance for hip intra-articular injections is essential [54]. Ultrasound guidance is advantageous for treating muscle injuries, particularly when deep or small muscles are involved [55]. Furthermore, it is worth stressing again that ultrasound guidance allows for the visualization of the relationship between the injured muscle and the surrounding nerves and vessels [55]. The literature supports that intra-articular knee injections can be performed without ultrasound guidance [54,56] and studies have shown high accuracy rates even in blind intra-articular knee injections [54]. However, the experts agreed that ultrasound guidance is recommended in cases of complicated knee anatomy.

### 4.8. Limitations of the Study

This study has some limitations. To begin, despite the fact that the physicians recruited here can be considered authorities in the field of musculoskeletal pain medicine (in this regard, they are either specialists in Physical and Rehabilitation Medicine or Rheumatologists) their precise level of expertise in collagen injection has not been quantified. For example, we did not collect data on the average number of injections performed by the Panelists. Second, our research was carried out exclusively within the framework of a national (Italian) scenario. Given that physicians from different countries might provide different responses caution seems appropriate in generalizing our findings to the global population of physicians dealing with musculoskeletal pain.

## 5. Conclusions

This Delphi study aimed to gather consensus on the modalities of execution of porcine collagen injections for different anatomical districts. Mainly, the frequency of injections. The possibility of combining collagen and other drugs in the same session. and several other aspects concerning collagen injections for the treatment of musculoskeletal disorders for which clear indications in the literature are lacking. We believe that this work provides insights into the current practice of porcine collagen injection procedures and represents an initial step toward establishing an informed clinical practice.

However, this process highlighted also that collagen injection modalities are still surrounded by many uncertainties, especially in specific contexts such as the possibility of combining collagen and anesthetics, HA, or steroids. The management of infiltrative therapy in the presence of inflammation and the optimal frequency and number of collagen injections needed to achieve a therapeutic effect in different anatomical districts and conditions. Hence, our research effectively underscores the necessity for further investigation into the clinical effectiveness of porcine collagen injections in alleviating pain and enhancing function in musculoskeletal disorders. Awaiting the results of clinical efficacy in an increasing number of human trials (RCTs), the current study may provide useful, albeit provisional, guidance to clinicians involved in musculoskeletal pain management.

## Figures and Tables

**Table 1:** Categories and their statements developed by the Steering Committee.

Categories	Number of Statements
Execution modalities of collagen injections in musculoskeletal disorders: e.g., tendinopathies, OA, myofascial pain).	8
2. Collagen injections in the treatment of joint pathologies with signs of active inflammation at the joint level (intra-articular, both in large and small joints).	2
3. Collagen injections in the treatment of joint pathologies in the absence of signs of inflammation at the joint level (intra-articular, both in large and small joints).	3
4. Collagen injections in the treatment of inflammatory or degenerative pathologies affecting various joint structures/soft tissues (tendons, ligaments, capsules, etc.).	6
5. Efficacy and safety of intra-articular collagen injections.	3
6. Efficacy and safety of collagen injections performed via extra-articular routes (periarticular, peri-tendinous, etc.).	7
7. Indication for performing with and without ultrasound guidance.	7

**Table 2:** Results of the Delphi survey for each statement belonging to category 1. Source: Original. Consensus: Panelists scoring their agreement with the eight statements.

Sum (%)		Weighted Average					Analyses					
1 + 2 L-LSV	4 + 5 H-LSV	1	2	3	4	5	HPD	Delta (%) LSVs	Delta WA Value	WA Value	*p* Value	Statement
13.04	30.43	0.000	0.130	1.696	0.696	0.652	3.00	17.39	0.17	3.17	0.1309	1.1
4.35	65.22	0.000	0.043	0.913	1.217	1.739	3.00	60.87	0.91	3.91	0.0003	1.2
8.70	82.61	0.000	0.087	0.261	1.391	2.391	3.00	73.91	1.13	4.13	0.0001	1.3
0.00	78.26	0.000	0.000	0.652	1.217	2.391	3.00	78.26	1.26	4.26	0.0001	1.4
0.00	100.00	0.000	0.000	0.000	0.870	3.913	3.00	100.00	1.78	4.78	0.0001	1.5
0.00	100.00	0.000	0.000	0.000	0.000	5.000	3.00	100.00	2.00	5.00	0.0001	1.6
0.00	86.96	0.000	0.000	0.391	1.739	2.174	3.00	86.96	1.30	4.30	0.0001	1.7
4.35	86.96	0.000	0.043	0.261	1.739	2.174	3.00	82.61	1.22	4.22	0.0001	1.8

Note: The number (1, 2, 3, 4 and 5) in the caption defines the relative Likert scores. Sum of percentage (Sum (%) of Low-Level of Likert Scale Values (L-LSV). High-Level of Likert Scale Values (V-LSV). Hypothetical Parametric Distribution (HPD). Variation in percents between (L-LSV) and (V-LSV). Variation in WA and PHD (Delta WA Value). Weight Average Value of statement (WA Value). Result of Wilcoxon test for the statement (*p* Value) and Result of Agreement in statement.

**Table 3:** Results of the Delphi survey for each statement belonging to category 2. Source: Original. Consensus: Panelists scoring their agreement with the two statements.

Sum (%)		Weighted Average					Analyses					
1 + 2 L-LSV	4 + 5 H-LSV	1	2	3	4	5	HPD	Delta (%) LSV	Delta WA Value	WA Value	*p* Value	Statement
52.17	30.43	0.043	0.478	0.522	0.696	0.652	3.00	−21.74	−0.61	2.39	0.7232	2.1
17.39	43.48	0.087	0.087	1.174	1.043	0.870	3.00	26.09	0.26	3.26	0.2183	2.2

Note: The number (1, 2, 3, 4 and 5) in the caption defines the relative Likert scores. Sum of percentage (Sum (%) of Low-Level of Likert Scale Values (L-LSV). High-Level of Likert Scale Values (V-LSV). Hypothetical Parametric Distribution (HPD). Variation in percents between (L-LSV) and (V-LSV). Variation in WA and PHD (Delta WA Value). Weight Average Value of statement (WA Value). Result of Wilcoxon test for the statement (*p* Value) and Result of Agreement in a statement.

**Table 4:** Results of the Delphi survey for each statement belonging to category 3. Source: Original. Consensus: Panelists scoring their agreement with the two statements.

Sum (%)		Weighted Average					Analyses					
1 + 2 L-LSV	4 + 5 H-LSV	1	2	3	4	5	HPD	Delta (%) LSV	Delta WA Value	WA Value	*p* Value	Statement
0.00	91.30	0.000	0.000	0.261	1.391	2.826	3.00	91.30	1.48	4.48	0.0001	3.1
4.35	95.65	0.000	0.043	0.000	1.739	2.609	3.00	91.30	1.39	4.39	0.0001	3.2
4.35	91.30	0.000	0.043	0.130	1.565	2.609	3.00	86.96	1.35	4.35	0.0001	3.3

Note: The number (1, 2, 3, 4 and 5) in the caption defines the relative Likert scores. Sum of percentage (Sum (%) of Low-Level of Likert Scale Values (L-LSV). High-Level of Likert Scale Values (V-LSV). Hypothetical Parametric Distribution (HPD). Variation in percents between (L-LSV) and (V-LSV). Variation in WA and PHD (Delta WA Value). Weight Average Value of statement (WA Value). Result of Wilcoxon test for the statement (*p* Value) and Result of Agreement in a statement.

**Table 5:** Results of the Delphi survey for each statement belonging to category 4. Source: Original. Consensus: Panelists scoring their agreement with the six statements.

Sum (%)		Weighted Average					Analyses					
1 + 2 L-LSV	4 + 5 H-LSV	1	2	3	4	5	HPD	Delta (%) LSV	Delta WA Value	WA Value	*p* Value	Statement
13.04	52.17	0.000	0.130	1.043	1.217	1.087	3.00	39.13	0.48	3.48	0.0110	4.1
0.00	95.65	0.000	0.000	0.130	1.565	2.826	3.00	95.65	1.52	4.52	0.0001	4.2
13.04	65.22	0.000	0.130	0.652	1.217	1.739	3.00	52.17	0.74	3.74	0.0014	4.3
0.00	78.26	0.000	0.000	0.652	1.913	1.522	3.00	78.26	1.09	4.09	0.0001	4.4
4.35	73.91	0.000	0.043	0.652	1.913	1.304	3.00	69.57	0.91	3.91	0.0001	4.5
8.70	56.52	0.000	0.087	1.043	1.565	0.870	3.00	47.83	0.57	3.57	0.0041	4.6

Note: The number (1, 2, 3, 4 and 5) in the caption defines the relative Likert scores. Sum of percentage (Sum (%) of Low-Level of Likert Scale Values (L-LSV). High-Level of Likert Scale Values (V-LSV). Hypothetical Parametric Distribution (HPD). Variation in percents between (L-LSV) and (V-LSV). Variation in WA and PHD (Delta WA Value). Weight Average Value of statement (WA Value). Result of Wilcoxon test for the statement (*p* Value) and Result of Agreement in a statement.

**Table 6:** Results of the Delphi survey for each statement belonging to category 5. Source: Original. Consensus: Panelists scoring their agreement with the three statements.

Sum (%)		Weighted Average					Analyses					
1 + 2 L-LSV	4 + 5 H-LSV	1	2	3	4	5	HPD	Delta (%) LSV	Delta WA Value	WA Value	*p* Value	Statement
0.00	100.00	0.000	0.000	0.000	1.391	3.261	3.00	100.00	1.65	4.65	0.0001	5.1
0.00	100.00	0.000	0.000	0.000	1.913	2.609	3.00	100.00	1.52	4.52	0.0001	5.2
8.70	82.61	0.000	0.087	0.261	1.565	2.174	3.00	73.91	1.09	4.09	0.0001	5.3

Note: The number (1, 2, 3, 4 and 5) in the caption defines the relative Likert scores. Sum of percentage (Sum (%) of Low-Level of Likert Scale Values (L-LSV). High-Level of Likert Scale Values (V-LSV). Hypothetical Parametric Distribution (HPD). Variation in percents between (L-LSV) and (V-LSV). Variation in WA and PHD (Delta WA Value). Weight Average Value of statement (WA Value). Result of Wilcoxon test for the statement (*p* Value) and Result of Agreement in a statement.

**Table 7:** Results of the Delphi survey for each statement belonging to category 6. Source: Original. Consensus: Panelists scoring their agreement with the six statements.

Sum (%)		Weighted Average					Analyses					
1 + 2 L-LSV	4 + 5 H-LSV	1	2	3	4	5	HPD	Delta (%) LSV	Delta WA Value	WA Value	*p* Value	Statement
4.35	82.61	0.000	4.348	13.043	34.783	47.826	3.00	78.26	1.22	4.22	0.0001	6.1
0.00	52.17	0.000	0.000	47.826	26.087	26.087	3.00	52.17	0.78	3.78	0.0005	6.2
4.35	65.22	0.000	4.348	30.435	39.130	26.087	3.00	60.87	0.83	3.83	0.0003	6.3
21.74	52.17	4.348	17.391	26.087	34.783	17.391	3.00	30.43	0.26	3.26	0.1009	6.4
30.43	43.48	0.000	30.435	26.087	21.739	21.739	3.00	13.04	0.04	3.04	0.1232	6.5
21.74	56.52	8.696	13.043	21.739	43.478	13.043	3.00	34.78	0.26	3.26	0.1640	6.6
30.43	34.78	8.696	21.739	34.783	21.739	13.043	3.00	4.35	−0.13	2.87	0.7817	6.7

Note: The number (1, 2, 3, 4 and 5) in the caption defines the relative Likert scores. Sum of percentage (Sum (%) of Low-Level of Likert Scale Values (L-LSV). High-Level of Likert Scale Values (V-LSV). Hypothetical Parametric Distribution (HPD). Variation in percents between (L-LSV) and (V-LSV). Variation in WA and PHD (Delta WA Value). Weight Average Value of statement (WA Value). Result of Wilcoxon test for the statement (*p* Value) and Result of Agreement in a statement.

**Table 8:** Results of the Delphi survey for each statement belonging to category 7. Source: Original. Consensus: Panelists scoring their agreement with the seven statements.

Sum (%)		Weighted Average					Analyses					
1 + 2 L-LSV	4 + 5 H-LSV	1	2	3	4	5	HPD	Delta (%) LSV	Delta WA Value	WA Value	*p* Value	Statement
0.00	95.65	0.000	0.000	0.130	0.522	4.130	3.00	95.65	1.78	4.78	0.0001	7.1
0.00	95.65	0.000	0.000	0.130	1.217	3.261	3.00	95.65	1.61	4.61	0.0001	7.2
0.00	100.00	0.000	0.000	0.000	1.217	3.478	3.00	100.00	1.70	4.70	0.0001	7.3
4.35	86.96	0.000	0.043	0.261	1.217	2.826	3.00	82.61	1.35	4.35	0.0001	7.4
0.00	95.65	0.000	0.000	0.130	0.174	4.565	3.00	95.65	1.87	4.87	0.0001	7.5
4.35	73.91	0.000	0.043	0.652	0.522	3.043	3.00	69.57	1.26	4.26	0.0001	7.6
0.00	95.65	0.000	0.000	0.130	1.565	2.826	3.00	95.65	1.52	4.52	0.0001	7.7

Note: The number (1, 2, 3, 4 and 5) in the caption defines the relative Likert scores. Sum of percentage (Sum (%) of Low-Level of Likert Scale Values (L-LSV). High-Level of Likert Scale Values (V-LSV). Hypothetical Parametric Distribution (HPD). Variation in percents between (L-LSV) and (V-LSV). Variation in WA and PHD (Delta WA Value). Weight Average Value of statement (WA Value). Result of Wilcoxon test for the statement (*p* Value) and Result of Agreement in a statement.

## Data Availability

The raw data supporting the conclusions of this article will be made available by the authors on request.

## Appendix A

Delphi panel of Italian experts and their region of work:

Aluena Battaglioli (Toscana); Federica Bertolucci (Toscana); Lucia Calbucci (Emilia-Romagna); Viviana Colantonio (Lombardia); Bruno Corrado (Campania); Alessandro De Sire (Calabria); Paolo di Benedetto (Friuli-Venezia Giulia); Giuseppe Falcone (Toscana); Giacomo Farì (Puglia); Paola Galligioni (Veneto); Nicole Lonoce (Toscana); Giorgio Mariani (Emilia-Romagna); Edoardo Milano (Piemonte); Annalisa Orioli (Toscana); Benedetta Panni (Lombardia); Teresa Paolucci (Abruzzo); Vincenzo Ricci (Lombardia); Paola Rodriguez (Toscana); Maria Teresa Pereira Ruiz (Liguria); Federico Salvò (Lombardia); Lucrezia Tognolo (Veneto); Francesca Uboldi (Lombardia); Nikoleta Vaso (Lombardia); Alen Zabotti (Friuli-Venezia Giulia).

The above-mentioned physicians/scientists demonstrated proven clinical activity within the National Health Service (SSN), as documented by their internal records of activities performed, or demonstrated their professional academic achievements through indexed publications in the field of “Musculoskeletal Disorders.” There were no conflicts of interest between the panel members and the selection committee.
